# A Novel Compound Ligusticum Cycloprolactam Alleviates Neuroinflammation After Ischemic Stroke via the FPR1/NLRP3 Signaling Axis

**DOI:** 10.1111/cns.70158

**Published:** 2024-12-09

**Authors:** Juan Gao, Gang Su, Jifei Liu, Jinyang Song, Wei Chen, Miao Chai, Xiaodong Xie, Manxia Wang, Junxi Liu, Zhenchang Zhang

**Affiliations:** ^1^ Department of Neurology, The Second Hospital & Clinical Medical School Lanzhou University Lanzhou Gansu China; ^2^ Institute of Genetics, School of Basic Medical Sciences Lanzhou University Lanzhou Gansu China; ^3^ Chinese Academy of Sciences Key Laboratory of Chemistry of Northwestern Plant Resources and Key Laboratory for Natural Medicine of Gansu Province, Lanzhou Institute of Chemical Physics Chinese Academy of Sciences Lanzhou Gansu China

**Keywords:** FPR1, ischemic stroke, LIGc, macrophages, microglia, neuroinflammation, NLRP3

## Abstract

**Background:**

Microglia/macrophages, as pivotal immune cells in the central nervous system (CNS), play a critical role in neuroinflammation associated with ischemic brain injury. Targeting their activation through pharmacological interventions represents a promising strategy to alleviate neurological deficits, thereby harboring significant implications for the prevention and treatment of ischemic stroke. Ligusticum cycloprolactam (LIGc), a novel monomeric derivative of traditional Chinese medicine, has shown potential as a therapeutic agent; however, its specific role in cerebral ischemic injury remains unclear.

**Methods:**

In vitro experiments utilized lipopolysaccharide (LPS)‐induced inflammation models of RAW264.7 cells and primary mouse microglia. In vivo studies employed LPS‐induced neuroinflammation models in mice and a transient middle cerebral artery occlusion (tMCAO) mouse model to evaluate the impact of LIGc on neuroinflammation and microglia/macrophage phenotypic alterations. Further elucidation of the molecular mechanisms underlying these effects was achieved through RNA‐Seq analyses.

**Results:**

LIGc exhibited the capacity to attenuate LPS‐induced production of pro‐inflammatory markers in macrophages and microglia, facilitating their transition to an anti‐inflammatory phenotype. In models of LPS‐induced neuroinflammation and tMCAO, LIGc ameliorated pathological behaviors and neurological deficits while mitigating brain inflammation. RNA‐seq analyses revealed formyl peptide receptor 1 (FPR1) as a critical mediator of LIGc's effects. Specifically, FPR1 enhances the pro‐inflammatory phenotype of microglia/macrophages and inhibits their anti‐inflammatory response by upregulating NLR family pyrin domain protein 3 (NLRP3) inflammasomes, thus aggravating inflammatory processes. Conversely, LIGc exerts anti‐inflammatory effects by downregulating the FPR1/NLRP3 signaling axis. Furthermore, FPR1 overexpression or NLRP3 agonists reversed the effects of LIGc observed in this study.

**Conclusion:**

Our findings suggest that LIGc holds promise in improving ischemic brain injury and neuroinflammation through modulation of microglia/macrophage polarization. Mechanistically, LIGc attenuates the pro‐inflammatory phenotype and promotes the anti‐inflammatory phenotype by targeting the FPR1/NLRP3 signaling pathway, ultimately reducing inflammatory responses and mitigating neurological damage.

## Graphical abstract

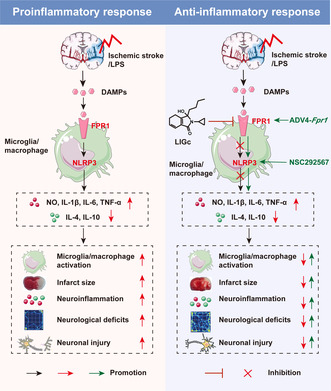

## 1 Introduction

Ischemic stroke constitutes approximately 71% of all stroke cases globally [1, 2], leading to irreversible neurological dysfunction within the CNS [3], and emerging as a major cause of disability and mortality worldwide [4]. Current treatments such as tissue plasminogen activator rt‐PA thrombolysis and mechanical thrombectomy using retrievable stents are established methods to restore blood flow postischemia [5, 6]. However, ischemia/reperfusion (I/R) injury exacerbates brain tissue damage, and rt‐PA is limited by strict time windows, contraindications, and the risk of symptomatic cerebral hemorrhage [7, 8]. Therefore, uncovering underlying mechanisms, identifying therapeutic targets, and developing more effective therapies hold crucial clinical significance.

After ischemic stroke, disruption of the local blood supply initiates a cascade of pathological changes [9, 10, 11], with the inflammatory response playing a pivotal role in its pathophysiology and its exacerbation during I/R injury [12]. Resident microglia and invasive macrophages occupy crucial positions in neuroinflammation following ischemic stroke [13, 14, 15]. These cells undergo morphological alterations and express common antigens, enabling them to phagocytose and adopt either pro‐inflammatory or anti‐inflammatory phenotypes [16]. Pro‐inflammatory markers, such as CD16, CD86, and inducible nitric oxide synthase (iNOS) [17], regulate gene expression related to inflammation, leading to the production of cytokines including tumor necrosis factor‐α (TNF‐α), interleukin‐6 (IL‐6), IL‐1β, and nitric oxide (NO), thereby augmenting neuronal damage [12]. Conversely, anti‐inflammatory markers, comprising IL‐4, IL‐10, and CD206, facilitate debris clearance, tissue regeneration, and attenuate inflammatory responses, thus exerting neuroprotective effects [18]. The activation states of microglia/macrophages—pro‐inflammatory and anti‐inflammatory—coexist and can transition between each other during the progression and management of CNS diseases [19]. Therefore, interventions aimed at redirecting microglia/macrophage activation toward an anti‐inflammatory phenotype could potentially enhance outcomes in ischemic stroke.

FPR1 belongs to the G protein‐coupled receptor (GPCR) superfamily [20, 21] and plays a critical role in modulating inflammatory responses triggered by microbial infections or tissue damage [22]. Excessive activation of intracellular NLRP3 inflammasomes due to FPR1‐mediated neuroinflammation in microglia/macrophages is closely linked to ischemic brain injury [23]. Studies have demonstrated that FPR1 agonists can activate NLRP3 inflammasomes in microglia, leading to damage to dopaminergic neurons [24]. Knockout of the *Fpr1* gene has been shown to downregulate NLRP3 inflammasome signaling, thereby mitigating chronic lung transplant rejection [25]. However, the specific impact of FPR1 on neuroinflammatory responses following cerebral ischemia through the regulation of NLRP3 inflammasome activation remains unclear and warrants further investigation.

**FIGURE 1:**
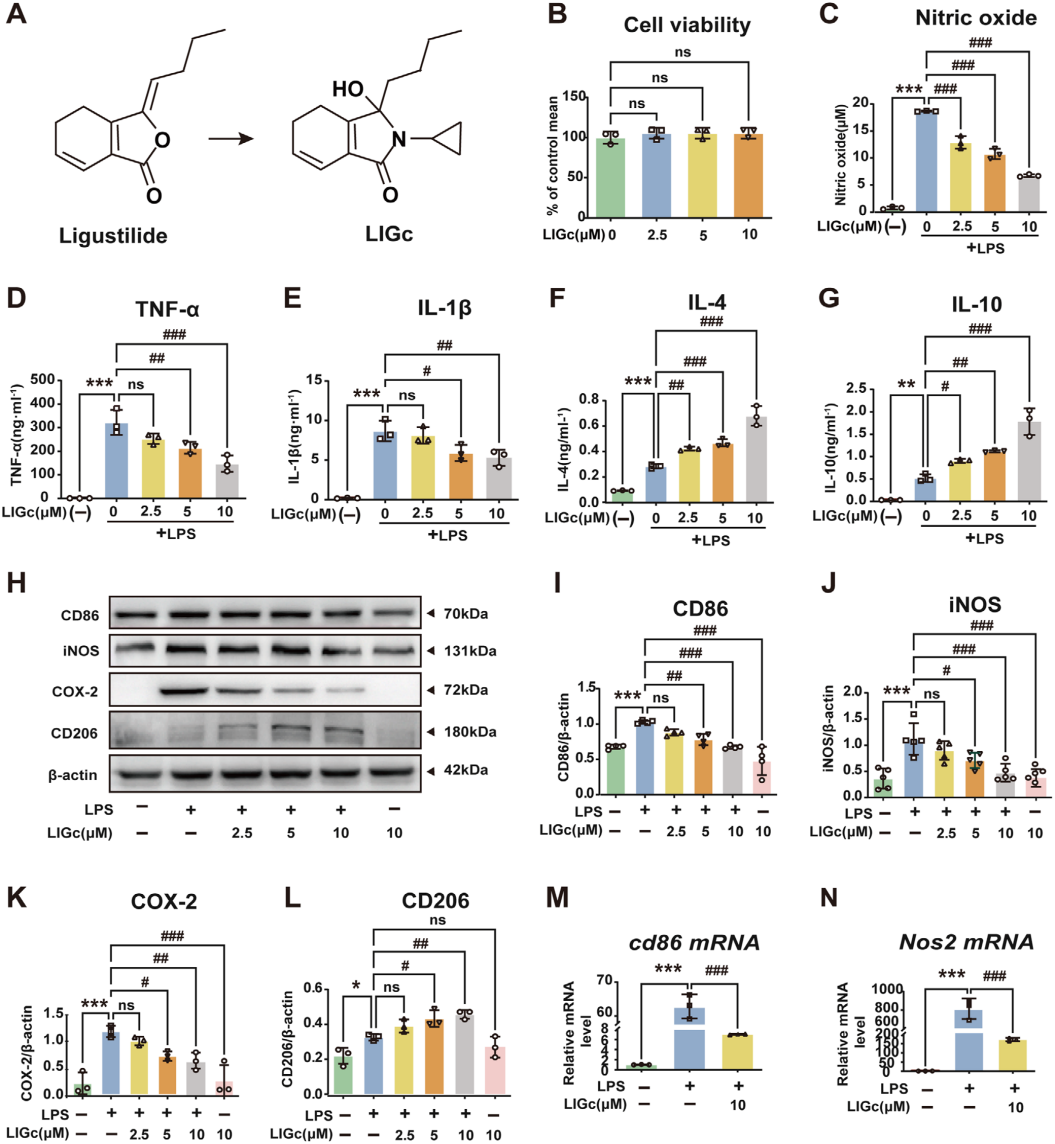
The impact of LIGc on the LPS‐induced transformation of RAW264.7 cells toward a pro‐inflammatory phenotype, alongside its promotion of anti‐inflammatory marker expression. (A) Molecular structures of ligustilide and LIGc. (B) Cytotoxicity evaluation of LIGc at various concentrations (0, 2.5, 5, 10 μM) on RAW264.7 cells over 24 h. (C–G) Griess and ELISA methods were used to quantify pro‐inflammatory cytokines (NO, TNF‐α, IL‐1β) and anti‐inflammatory cytokines (IL‐4, IL‐10). (H–L) Western blot analysis was employed to assess the expression levels of pro‐inflammatory proteins CD86, iNOS, and COX‐2 as well as the anti‐inflammatory protein CD206. (M, N) qRT‐PCR was utilized to evaluate the impact of LIGc intervention on the expression of pro‐inflammatory markers *cd86* and *Nos2* genes. Results are presented as mean ± SD (*n* ≥ 3 independent experiments). Compared to the control group, *p < 0.05, **p < 0.01, ***p < 0.001; compared to the LPS group, #*p* < 0.05, ##*p* < 0.01, ###*p* < 0.001.

Ligustilide (C_12_H_14_O_2_) is the principal active compound found in the volatile oil of rhizomes from *Chuanxiong* and *Angelica sinensis*, traditional Chinese medicines belonging to the Umbelliferae family [26]. It exhibits notable anti‐inflammatory, antioxidative stress, apoptosis inhibition, and neuroprotective properties [27]. However, its chemical instability has posed challenges for the development and application of pharmaceutical products containing this compound [28]. LIGc (Figure 1A), a colorless needle‐like crystal derived from ligustilide (Patent number 201810730744.7), addresses this issue by enhancing ligustilide's chemical stability while preserving its activity and safety, and moreover, LIGc is easily able to penetrate the blood–brain barrier [29]. Despite its potential, little is known about the role of this novel Chinese medicine derivative in the CNS. Our previous research indicated that LIGc reduces BV2 microglia‐mediated inflammatory responses, thereby decreasing neurotoxicity toward HT‐22 hippocampal nerve cells [30]. However, its precise impact on neuroinflammation has not been comprehensively elucidated through in vivo experiments and reverse verification. In this study, we employed RNA‐Seq to explore the molecular mechanisms underlying LIGc's effects on neuroinflammation. Our findings demonstrate that LIGc modulates the inflammatory phenotype of RAW264.7 macrophages and primary microglia, improves pathological behavior and neurological dysfunction in neuroinflammatory and tMCAO mouse models, and thus attenuates brain inflammatory responses. Mechanistically, LIGc downregulates the FPR1/NLRP3 signaling pathway, thereby suppressing the production of inflammatory mediators and exerting neuroprotective effects.

## 2 Methods

### 2.1 Reagents

LIGc was synthesized following the method previously reported by Professor Liu Junxi at the Lanzhou Institute of Chemical Physics, Chinese Academy of Sciences [29], with purity > 95% (Figure S1). All other reagents were sourced commercially and were of the highest purity available.

### 2.2 Cell Culture

RAW264.7 mouse macrophages (Pricella, Wuhan, China) were cultured in high‐glucose DMEM (Gibco, Grand Island, NY, USA) supplemented with 10% heat‐inactivated fetal bovine serum, 100 μg/mL streptomycin, and 100 U/mL penicillin. Cells were maintained in a cell culture incubator (Heal Force, Shanghai, China) at 37°C with 5% CO_2_ and 95% air.

Primary mouse microglia were isolated as previously described [31]. Following the removal of meninges, digestion, mincing, centrifugation, and collection of pellets from the forebrains of neonatal Kunming mice (within 3 days of birth), the pellets were inoculated into a 25 cm [2] cell culture flask previously coated with poly‐l‐lysine solution (Solarbio, Beijing, China). The culture medium was refreshed on the third and sixth days. Upon reaching confluence between days 10 and 14, microglia were isolated using mild trypsin digestion and seeded into 96‐well or 24‐well plates. Immunofluorescence staining with ionized calcium‐binding adaptor molecule 1 (Iba1) antibody (Proteintech Group; Cat. No. 81728‐1‐RR) confirmed microglial purity (> 95%), allowing subsequent experimental procedures.

### 2.3 Cell Model Construction

Microglia or macrophages were treated with varying concentrations of LIGc (2.5, 5, 10 μM) for 2 h. Subsequently, 100 ng/mL of LPS (Sigma‐Aldrich; Cat No. L2630) was added for either 6 or 12 h to assess the expression of pro‐inflammatory and anti‐inflammatory markers at the gene or protein levels.

### 2.4 Cell Viability Assay

Cell viability was assessed using the Cell Counting Kit‐8 (CCK‐8) (Boster, Wuhan, China). RAW264.7 cells and primary mouse microglia were seeded into 96‐well plates and treated with varying concentrations of LIGc (0, 2.5, 5, 10 μM) or DMSO (0.2%, v/v). After 24 h of treatment, 10 μL of CCK‐8 reagent was added to each well and incubated at 37°C for 1 h. Absorbance at 450 nm was measured using a SynergyNeo2 full‐featured microplate reader (Agilent, CA, USA).

### 2.5 NO Determination

NO production was evaluated by measuring nitrite levels in cell culture supernatants. Supernatants obtained from RAW264.7 cells and primary mouse microglia were collected, and nitrite concentrations were determined using an NO assay kit (Jiancheng Bioengineering Institute, Nanjing, China), following the manufacturer's protocol. Absorbance at 550 nm was measured using a Synergy Neo2 microplate reader. NO concentrations were quantified using a sodium nitrite standard solution.

### 2.6 Enzyme‐Linked Immunosorbent Assay (ELISA)

The impact of LIGc on cytokine protein levels was assessed using ELISA. Cell supernatants or mouse brain tissues were collected, and the concentrations of TNF‐α (Cat. No. E‐EL‐M3063), IL‐1β (Cat. No. E‐EL‐M0037), IL‐4 (Cat. No. E‐EL‐M0043), and IL‐10 (Cat. No. E‐EL‐M0046) were determined using commercial ELISA kits (Elabscience, Wuhan, China). Absorbance was measured at 450 nm using a SynergyNeo2 microplate reader.

### 2.7 Quantitative RT‐PCR (qRT‐PCR)

Total RNA from RAW264.7 cells was extracted using Trizol reagent (Mei5bio; Cat No. MF736‐01). The RNA was reverse transcribed into cDNA using SweScript All‐in‐One Blue RT SuperMix for qPCR (Servicebio; Cat No. G3329). Real‐time PCR was conducted with SYBR Green qPCR Master Mix (Servicebio; Cat No. G3326) on the ABI QuantStudio3 Real‐Time PCR System (Carlsbad, USA). Cycle threshold (*C*_t_) values were normalized to *β‐actin* levels, and fold changes between control and treatment groups were determined using the 2^−ΔΔCt^ method. The primer sequences used are listed in Table 1.

**TABLE 1:** Primers for qPCR.

Gene	Forward primers (5′–3′)	Reverse primers (5′–3′)
*Nos2*	GTTCTCAGCCCAACAATACAAA	GTGGACGGGTCGATGTCAC
*cd86*	AACGTATTGGAAGGAGATTACAGCT	CCTGCTAGGCTGATTCGGCT
*Fpr1*	TGCCGTCACATTTGTCCTTG	TGGCAAAGTGGAAGTGAAGC
*Fpr2*	TTGGATCCTGGGCTCAAACT	TCGTAAAGGACGGCTGGAAT
*Lcn2*	ATGTCACCTCCATCCTGGTC	AACTGGTTGTAGTCCGTGGT
*Il‐6*	GACTGATGCTGGTGACAACC	AGACAGGTCTGTTGGGAGTG
*Pacsin1*	GAACAGCCTGCTGAATGAGG	CCACCCATGATCTGCTTGTG
*Retn1*	GAAGGCACAGCAGTCTTGAG	GCTGCTGTCCAGTCTATCCT
*β‐Actin*	GTGACGTTGACATCCGTAAAGA	GTAACAGTCCGCCTAGAAGCAC

### 2.8 Western Blot

Brain tissue or cells were lysed using RIPA lysate (Beyotime; Cat. P0013B). The protein concentration was determined with the BCA Protein Assay Kit (Beyotime; Cat. P0012S). Protein samples were separated by SDS‐PAGE and transferred onto PVDF membranes (Millipore, Massachusetts, USA). Following blocking with 5% skimmed milk powder for 1 h, membranes were incubated overnight at 4°C with primary antibodies, and subsequently incubated with horseradish peroxidase (HRP)‐conjugated secondary antibodies for 1 h at room temperature. Immunoreactive bands were visualized using a chemiluminescence imaging system (VILBER BIO IMAGING, Paris, France). Ratios of target protein to β‐actin or GAPDH were calculated. The antibodies used are listed below: anti‐CD86 (Santa Cruz; Cat. sc‐28347; 1:800), anti‐iNOS (Proteintech; Cat. 22226‐1‐AP; 1:1000), anti‐COX‐2 (Proteintech; Cat. 66351‐1‐Ig; 1:1000), anti‐CD206 (Proteintech; Cat. 18704‐1‐AP; 1:1000), anti‐FPR1 (Invitrogen; Cat. PA1‐41398; 1:1000), anti‐NLRP3 (Invitrogen; Cat. PA5‐79740; 1:1000), anti‐β‐actin (Proteintech; Cat. 66009‐1‐Ig, 20,536‐1‐AP; 1:5000), anti‐GAPDH (Proteintech; Cat. 60004‐1‐Ig; 1:5000), HRP‐labeled goat anti‐rabbit IgG (Proteintech; Cat. SA00001‐2; 1:4000), HRP‐labeled goat anti‐mouse IgG (Proteintech; Cat. SA00001‐1; 1:4000).

### 2.9 Immunofluorescence

Cells were washed with PBS, fixed with 4% paraformaldehyde (Sigma‐Aldrich, St. Louis, Missouri, USA) for 15 min, and permeabilized with 0.1% (v/v) Triton X‐100 (Sigma‐Aldrich, St. Louis, Missouri, USA) for 15 min. For mice, brains were perfused with PBS and 4% paraformaldehyde immediately after sacrifice, fixed with 4% paraformaldehyde for 24 h, and subsequently immersed in 30% sucrose solution until sinking to the bottom. Brain tissues were embedded in 100% OCT compound (SAKURA, USA), frozen at −80°C, and sectioned into 8 μm‐thick coronal sections using a microtome (CM1950, Leica, Germany). Cells or brain slices were blocked with 5% skimmed milk for 1 h, then incubated overnight at 4°C with anti‐CD86 primary antibody (Santa Cruz; Cat. sc‐28347; 1:300) and anti‐Iba1 primary antibody (Abcam; Cat. Ab178846; 1:500), followed by incubation with fluorescent conjugated secondary antibodies for 1 h in the dark. Nuclear staining was performed using DAPI Staining Solution (Beyotime; Cat. C1005). Images were captured using a research‐grade inverted microscopic imaging system (NIKON ECLIPSE Ti2‐E, Nikon, Japan).

### 2.10 Cell Transfection

The adenovirus vector (ADV4‐*Fpr1*), encoding the mouse *Fpr1* gene (NCBI ID: NM_013521.2), and the control vector were obtained from GenePharma (Shanghai, China). RAW264.7 cells or primary microglia were transduced with ADV4‐*Fpr1* or a negative control adenovirus for 24 h. Subsequently, cells were washed gently with cell culture medium containing 5% FBS and maintained in the same medium for an additional 48 h. Cell samples were collected and analyzed starting 72 h postinfection.

### 2.11 RNA‐Seq

RAW264.7 cells were incubated with 10 μM LIGc for 2 h and subsequently treated with 100 ng/mL LPS for 6 h. Total RNA was extracted and sequenced by BGI Genomics Co. Ltd. (Shenzhen, China). Gene fragment comparisons were conducted using HISAT2 (v2.0.4) [32]. DESeq2 (v1.4.5) was employed for differential expression analysis, with an adjusted *p* value threshold of 0.05. Genes with |Fold change value| ≥ 1.5 were considered significantly differentially expressed genes (DEGs). Gene expression profiles were analyzed using hierarchical clustering and volcano plots. Gene ontology (GO), Kyoto Encyclopedia of Genes and Genomes (KEGG) pathway enrichment analysis, and gene set enrichment analysis (GSEA) were performed on DEGs. The raw sequencing data have been deposited in the NCBI Sequence Read Archive (SRA) under accession number PRJNA1132545 (https://www.ncbi.nlm.nih.gov/sra/PRJNA1132545).

### 2.12 Molecular Docking

Molecular docking calculations were performed to investigate the interaction between LIGc and the crystal structure of FPR1. The mol2 format file of LIGc was generated using ChemDraw and Chem3D software, while the crystal structure of FPR1 (PDB ID:7EUO) was obtained from the PDB database [33]. Solvent molecules and ligands were removed from the crystal structure using PYMOL to produce a pdbqt format file. Docking simulations were conducted using AutoDock Vina software. A binding affinity of < −5.0 kcal/mol was considered indicative of favorable binding activity between the drug molecule and the protein.

### 2.13 Animals

Adult male Kunming mice (24 ± 2 g, aged 8–10 weeks) were obtained from the Experimental Animal Center of Lanzhou University (Lanzhou, China; Laboratory Animal Certificate: SCXK(Gan)2018‐0002). The animals were housed under controlled conditions with a temperature range of 20°C–25°C, a 12‐h light‐dark cycle, and relative humidity maintained between 40% and 60%. They had ad libitum access to standard laboratory feed and water. All experimental procedures involving animals were approved by the Ethics Committee of the Second Hospital & Clinical Medical School, Lanzhou University (Approval Number: D2022‐266). The experiments were conducted in compliance with the regulations outlined in the Chinese Management of Laboratory Animals and the Guidelines for the Care and Use of Experimental Animals of the National Academy of Sciences (http://oacu.od.nih.gov/regs/index.htm).

### 2.14 LPS‐Induced Neuroinflammatory Model

Twenty‐four mice were randomly divided into four groups (6 mice per group): the control group, LPS + vehicle group, LPS + LIGc (20 mg/kg) group, and LPS + LIGc (60 mg/kg) group. LIGc solution (20 or 60 mg/kg) or vehicle (0.5% CMC‐Na, Sigma‐Aldrich) was administered orally once daily at 8:30 am for four consecutive days. On the fourth day, mice received an intraperitoneal injection of LPS (Sigma‐Aldrich, 1 mg/kg) or saline 2 h after oral gavage. The open field test was conducted 4 h after the injection. After 24 h, mice were deeply anesthetized and euthanized, and their cerebral cortex was collected for further analysis.

### 2.15 Open Field Test

The exercise activity of mice was assessed using the open field test. Mice were gently positioned in the center of an open field arena (TECHMAN, Chengdu, China), and their movements were recorded using BAS‐100 software (TECHMAN Software Co. Ltd.). Each mouse was recorded for 5 min continuously. Parameters including distance covered, distance in center, time in center, time in corner, activity time, and rest time were analyzed.

### 2.16 tMCAO Model

Fifty‐six mice were randomly allocated into four groups: sham (12 mice), tMCAO + vehicle (13 mice), tMCAO + LIGc (20 mg/kg) (16 mice), and tMCAO + LIGc (60 mg/kg) (15 mice) groups. LIGc (20 and 60 mg/kg) was administered via oral gavage at 1, 24, and 48 h post‐I/R. Mice were anesthetized with isoflurane, and the right external and internal carotid arteries were surgically exposed via a mid‐neck incision. A monofilament nylon suture (Beijing Cinontech Co. Ltd., China) was gently advanced into the internal carotid artery via the external carotid artery to induce tMCAO. After 60 min, the suture was withdrawn to allow reperfusion. The sham group underwent the same procedure without insertion of the nylon suture. Following surgery, mice were placed under a heat lamp for 2 h to maintain body temperature. During the experiment, 6.67% of mice were excluded due to failed model induction or death.

### 2.17 Neurological Function Score

Neurological deficits were evaluated using the modified Longa method [34]: 0 points indicated no neurological dysfunction; 1 point denoted inability to fully extend the contralateral forepaw; 2 points indicated contralateral turning during walking; 3 points represented contralateral tilting while walking, signifying severe neurological dysfunction; and 4 points indicated inability to walk spontaneously and a significant decrease in consciousness level. Assessments were conducted by blinded observers who were unaware of the study design and group allocation.

### 2.18 Cerebral Infarction Volume Measurement

Cerebral infarction volume was assessed using 2,3,5‐triphenyltetrazolium chloride (TTC) staining 72 h postmodeling. Following euthanasia, mouse brain tissue was sectioned into 2‐mm‐thick coronal slices, which were then stained with a 2% (w/v) TTC solution (Solarbio; Cat. T8170) at 37°C for 15 min. The area of cerebral infarction in each section was quantified using ImageJ software. To mitigate the effects of brain edema, the infarct area of each brain slice was normalized to the noninfarcted contralateral side using the formula: infarct area (%) = (contralateral area − ipsilateral noninfarct area)/contralateral area × 100.

### 2.19 Neurobehavioral Tests

Corner test: The corner test was employed to assess sensorimotor asymmetry in mice [35]. Mice were placed facing into a 30° angle formed by two plates. Upon touching the bilateral whiskers against the corner, healthy mice exhibited random left or right turns, whereas mice with tMCAO tended to turn preferentially toward the nonparalyzed (right) side. Each mouse underwent 10 trials, and the frequency of right‐side turns was recorded.

Cylinder test: The cylinder test evaluated forelimb motor function impairment in mice [36]. Mice were positioned inside a transparent cylinder and observed as they explored by rearing up and touching the cylinder walls with their forelimbs. Mice with tMCAO showed a preference for using their nonparalyzed (right) forelimb. The forelimb impairment score (%) was calculated using the formula: (contralateral contacts +1/2 bilateral contacts)/total contacts × 100%.

### 2.20 Statistical Analysis

Data are presented as mean ± SD. Statistical analysis was performed using GraphPad Prism 9 software (GraphPad Software Inc., CA, USA). The Shapiro–Wilk test was used to assess normality, and the Brown‐Forsythe test was employed for testing variance homogeneity in multiple groups. Independent sample t‐tests were conducted for comparisons between two groups, while one‐way analysis of variance (ANOVA) or two‐way ANOVA followed by Tukey's post hoc tests were used for comparisons among multiple groups. Neurological function scores were analyzed using the Kruskal–Wallis test. A significance level of *p* < 0.05 was used to determine statistical significance.

## 3 Results

### 3.1 LIGc Modulates LPS‐Induced Inflammatory Response in RAW264.7 Cells

We investigated the effect of LIGc on the LPS‐induced inflammatory response in RAW264.7 cells in vitro. Initially, we assessed the impact of LIGc on cell viability using the CCK‐8 assay. It was observed that concentrations of LIGc below 10 μM had no significant effect on cell survival rates (Figure 1B). Subsequently, RAW264.7 cells were pretreated with LIGc (0, 2.5, 5, 10 μM) for 2 h followed by co‐treatment with LPS (100 ng/mL) for 12 h. Griess and ELISA assays revealed that LIGc dose‐dependently inhibited the production of NO (Figure 1C), TNF‐α (Figure 1D), and IL‐β (Figure 1E) in the cell supernatant induced by LPS, while enhancing the secretion of anti‐inflammatory cytokines IL‐4 (Figure 1F) and IL‐10 (Figure 1G), with optimal effects observed at 10 μM. Furthermore, western blot analysis demonstrated that LIGc attenuated the expression of pro‐inflammatory proteins CD86, iNOS, and cyclooxygenase 2 (COX‐2) in LPS‐stimulated RAW264.7 cells in a dose‐dependent manner (Figure 1H–K), and upregulated the expression of CD206 (Figure 1H,L). qRT‐PCR results indicated that LIGc suppressed the transcriptional levels of *cd86* and *Nos2* induced by LPS in RAW264.7 cells (Figure 1M,N). These findings collectively suggest that LIGc exhibits anti‐inflammatory properties by inhibiting pro‐inflammatory activation and promoting anti‐inflammatory responses in RAW264.7 macrophages.

### 3.2 LIGc Alleviates LPS‐Induced Inflammatory Response in Primary Microglia

**FIGURE 2:**
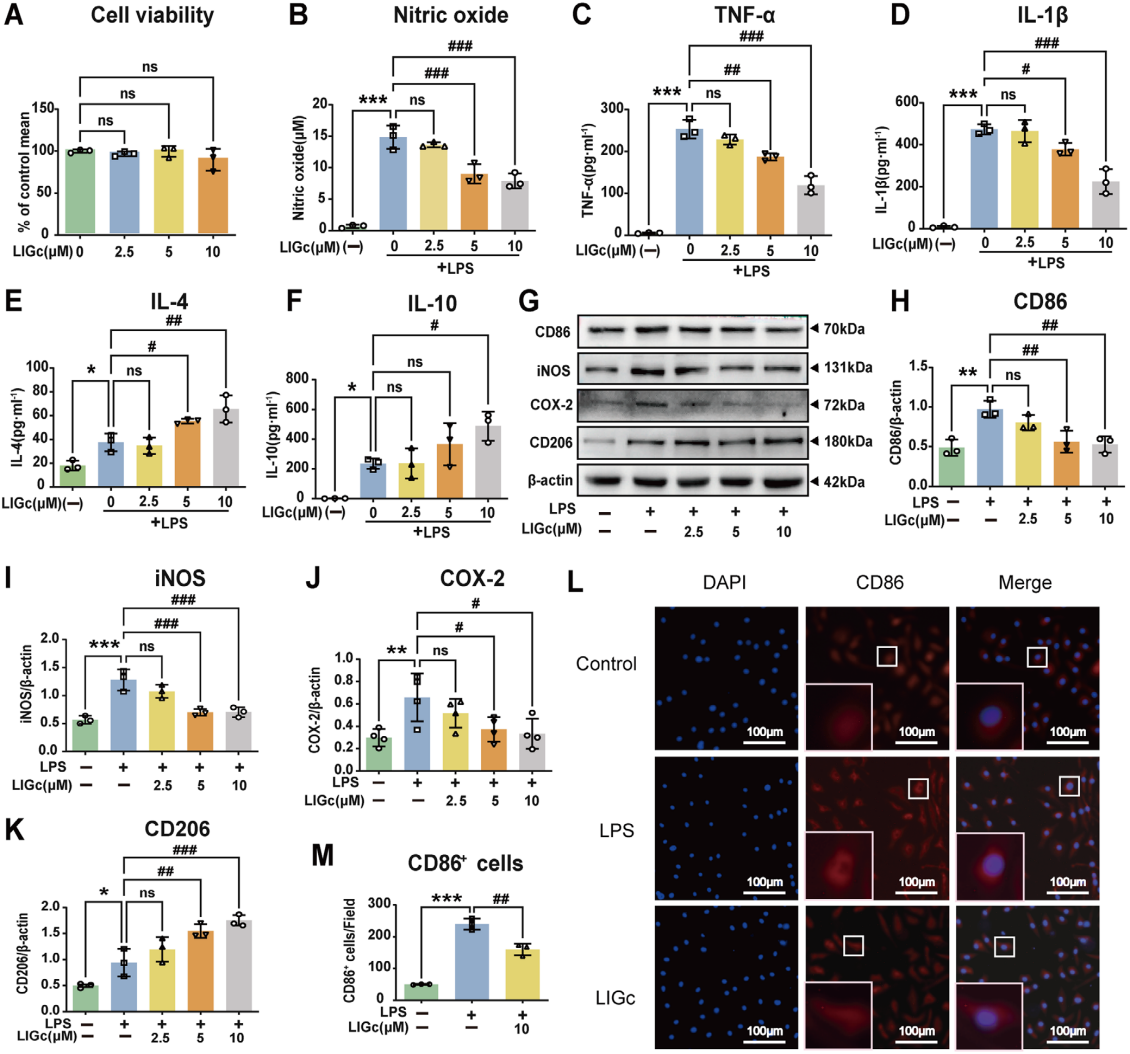
LIGc suppresses the LPS‐induced pro‐inflammatory phenotype of primary mouse microglia and promotes their transition to an anti‐inflammatory phenotype. (A) Primary microglia were exposed to gradient concentrations of LIGc (0, 2.5, 5, and 10 μM) for 24 h to assess cytotoxicity. (B–F) The Griess method or ELISA was employed to quantify levels of pro‐inflammatory cytokines NO, TNF‐α, and IL‐1β, as well as anti‐inflammatory cytokines IL‐4 and IL‐10 in the conditioned medium. (G–K) Western blot analysis was conducted to measure the expression of pro‐inflammatory proteins CD86, iNOS, and COX‐2, along with the anti‐inflammatory protein CD206. (L, M) Immunofluorescence staining was utilized to evaluate the impact of LIGc treatment on the expression of the pro‐inflammatory marker CD86. Data are presented as mean ± SD (*n* ≥ 3 independent experiments). Compared to the control group, *p < 0.05, **p < 0.01, ***p < 0.001; compared to the LPS group, #*p* < 0.05, ##*p* < 0.01, ###*p* < 0.001.

To validate the anti‐inflammatory effect of LIGc in primary microglia, we isolated microglia from the mouse cerebral cortex and assessed their purity using the microglia marker Iba1 (Figure S2). Cell viability assessed by CCK‐8 assay indicated that LIGc concentrations up to 10 μM had no significant impact on cell survival rates (Figure 2A). Following LIGc treatment, the levels of pro‐inflammatory factors NO (Figure 2B), TNF‐α (Figure 2C), and IL‐β (Figure 2D) in the cell supernatant were markedly reduced compared to the LPS‐stimulated group. Conversely, the release of anti‐inflammatory cytokines IL‐4 (Figure 2E) and IL‐10 (Figure 2F) was increased. Western blot analysis further revealed that LIGc suppressed the expression of pro‐inflammatory proteins CD86, iNOS, and COX‐2 in LPS‐induced primary microglia (Figure 2G–J), while enhancing the expression of CD206 (Figure 2G,K). Immunofluorescence staining additionally demonstrated decreased expression of CD86, a marker of pro‐inflammatory activation in primary microglia, following LIGc treatment (Figure 2L,M). These findings collectively indicate that LIGc attenuates the pro‐inflammatory response mediated by primary microglia.

### 3.3 LIGc Improves Motor Activity and Inflammation in LPS‐Induced Neuroinflammatory Mice

**FIGURE 3:**
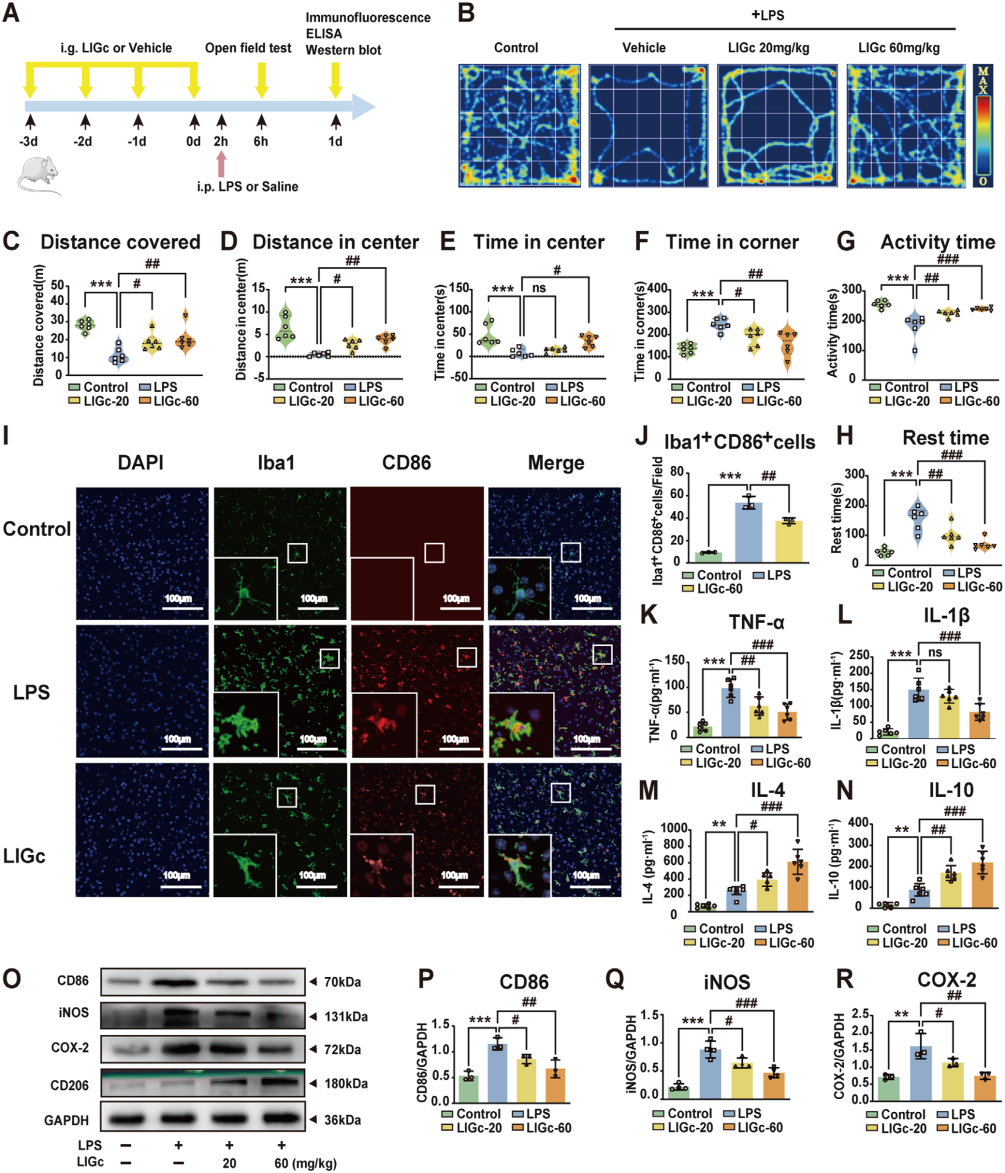
LIGc mitigates neuroinflammatory responses induced by LPS in mice. (A) Experimental design schematic. (B–H) The open field test assessed the impact of LIGc on behavioral metrics including distance covered, distance in center, time in center, time in corner, activity time, and rest time in mice stimulated by LPS. (I, J) Double‐staining immunofluorescence analysis evaluated the impact of LIGc on Iba1^+^CD86^+^ cell counts in the brains of neuroinflammatory mice. (K–N) ELISA quantified the levels of pro‐inflammatory mediators TNF‐α and IL‐1β as well as anti‐inflammatory cytokines IL‐4 and IL‐10 in the brains of neuroinflammatory mice following LIGc treatment. (O–R) Western blot analysis assessed the impact of LIGc on the expression of pro‐inflammatory proteins CD86, iNOS, and COX‐2, and anti‐inflammatory protein CD206 in neuroinflammatory mouse brains. Data are presented as mean ± SD (6 mice per group for B–H, K–N; 3 mice per group for I–J, O–R; *n* ≥ 3 independent experiments). Compared to the control group, **p < 0.01, ***p < 0.001; compared to the LPS group, #*p* < 0.05, ##*p* < 0.01, ###*p* < 0.001.

In light of LIGc's pronounced anti‐inflammatory effects observed in vitro, we established a mouse model of LPS‐induced neuroinflammation (Figure 3A) to investigate its impact on behavioral function and neuroinflammation in vivo. The open field test (Figure 3B) revealed that LIGc administered at doses of 20 or 60 mg/kg significantly ameliorated LPS‐induced alterations in several behavioral metrics, including distance covered (Figure 3C), distance in center (Figure 3D), time in center (Figure 3E), time in corner (Figure 3F), activity time (Figure 3G), and rest time (Figure 3H), indicating its potential to enhance motor activity and exploratory behavior in neuroinflammatory mice. Subsequent assessment of microglia/macrophage activation in the cerebral cortex of these mice showed that LIGc treatment attenuated the LPS‐induced increase in the number of Iba1^+^CD86^+^ cells, as evidenced by double‐staining immunofluorescence (Figure 3I,J). Further quantitative analysis of pro‐inflammatory and anti‐inflammatory phenotypes in cortical tissue supported LIGc's anti‐inflammatory effects. ELISA and western blot analyses demonstrated that LIGc significantly suppressed levels of pro‐inflammatory markers TNF‐α (Figure 3K), IL‐1β (Figure 3L), CD86 (Figure 3O,P), iNOS (Figure 3O,Q), and COX‐2 (Figure 3O,R) compared to the LPS group, while concurrently enhancing the expression of anti‐inflammatory markers IL‐4 (Figure 3M), IL‐10 (Figure 3N), and CD206 (Figure 3O, Figure S3). These findings collectively indicate that LIGc effectively improves both behavioral activities and inflammatory responses in LPS‐induced neuroinflammatory mice.

### 3.4 LIGc Alleviates Neurological Deficit and Inflammatory Response in tMCAO Mice

**FIGURE 4:**
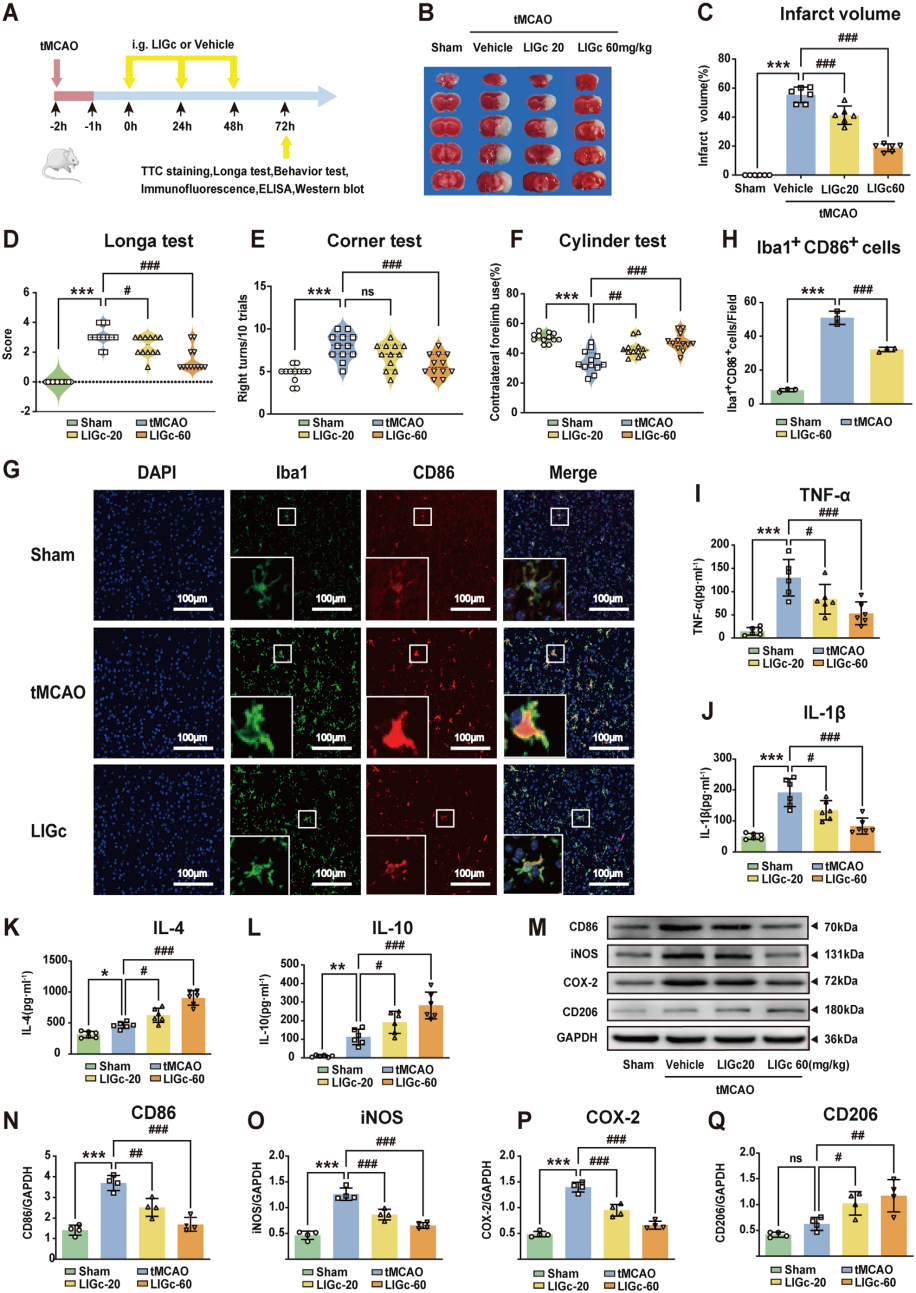
LIGc demonstrates efficacy in ameliorating cerebral ischemic injury and neuroinflammation in a tMCAO mouse model. (A) Experimental design schematic. (B, C) LIGc treatment significantly reduced cerebral infarction volume 72 h post‐tMCAO. (D–F) Neurological function was evaluated using the Longa test, corner test, and cylinder test to assess the impact of LIGc on tMCAO‐induced deficits. (G, H) Double‐staining immunofluorescence analysis assessed the impact of LIGc on Iba1^+^CD86^+^ cell counts in the infarcted cerebral cortex. (I–L) ELISA quantification revealed altered levels of pro‐inflammatory mediators TNF‐α, IL‐1β, and anti‐inflammatory cytokines IL‐4 and IL‐10 due to LIGc treatment. (M–Q) Western blot analysis showed modulation of pro‐inflammatory proteins CD86, iNOS, COX‐2, and anti‐inflammatory protein CD206 following LIGc administration. Results are presented as mean ± SD (6 mice per group for B–C, I–L; 12 mice per group for D–F; 3 mice per group for G–H, M–Q; *n* ≥ 3 independent experiments). Compared to the Sham group, *p < 0.05, **p < 0.01, ***p < 0.001; compared to the tMCAO group, #*p* < 0.05, ##*p* < 0.01, ###*p* < 0.001.

Secondary neurological impairment mediated by neuroinflammation is a significant factor contributing to injury following cerebral ischemia [15, 37]. Here, we investigated the therapeutic potential of LIGc in a mouse model of tMCAO (Figure 4A). The results of TTC staining (Figure 4B,C) revealed extensive infarction in the brains of tMCAO mice, whereas LIGc treatment dose‐dependently reduced infarct volume 3 days post‐tMCAO. To evaluate LIGc's impact on neurological deficits, we conducted the Longa test and behavioral tests. As depicted in Figure 4D, the Longa scores were significantly lower in the LIGc‐treated group compared to the tMCAO group, indicating improved neurological function with LIGc treatment. Additionally, in the corner test and cylinder test, tMCAO mice exhibited rightward turning tendencies (Figure 4E) and reduced use of affected limbs in the cylinder (Figure 4F), whereas LIGc treatment effectively ameliorated these sensorimotor asymmetries (Figure 4E,F). These findings collectively demonstrate that LIGc reduces cerebral infarction volume and improves neurological function in stroke mice. Given that overactivation of microglia/macrophages exacerbates poststroke neurological dysfunction, we assessed the expression of the pro‐inflammatory marker CD86 and microglia/macrophage‐specific cellular marker Iba1 via double‐staining immunofluorescence. Compared to the tMCAO group, LIGc treatment significantly reduced the number of Iba1^+^CD86^+^ cells (Figure 4G,H). Furthermore, ELISA and western blot analyses revealed elevated levels of pro‐inflammatory markers TNF‐α (Figure 4I), IL‐1β (Figure 4J), CD86 (Figure 4M,N), iNOS (Figure 4M,O), and COX‐2 (Figure 4M,P) in the cerebral cortex surrounding the infarct area 3 days post‐I/R in untreated mice, whereas LIGc treatment attenuated these increases. Additionally, LIGc treatment enhanced the expression of anti‐inflammatory markers IL‐4 (Figure 4K), IL‐10 (Figure 4L), and CD206 (Figure 4M,Q). Collectively, these results indicate that LIGc mitigates neuroinflammation and reduces cerebral ischemic damage in tMCAO mice.

### 3.5 RNA‐Seq Analysis Reveals That LIGc Suppresses the Expression of FPR1

**FIGURE 5:**
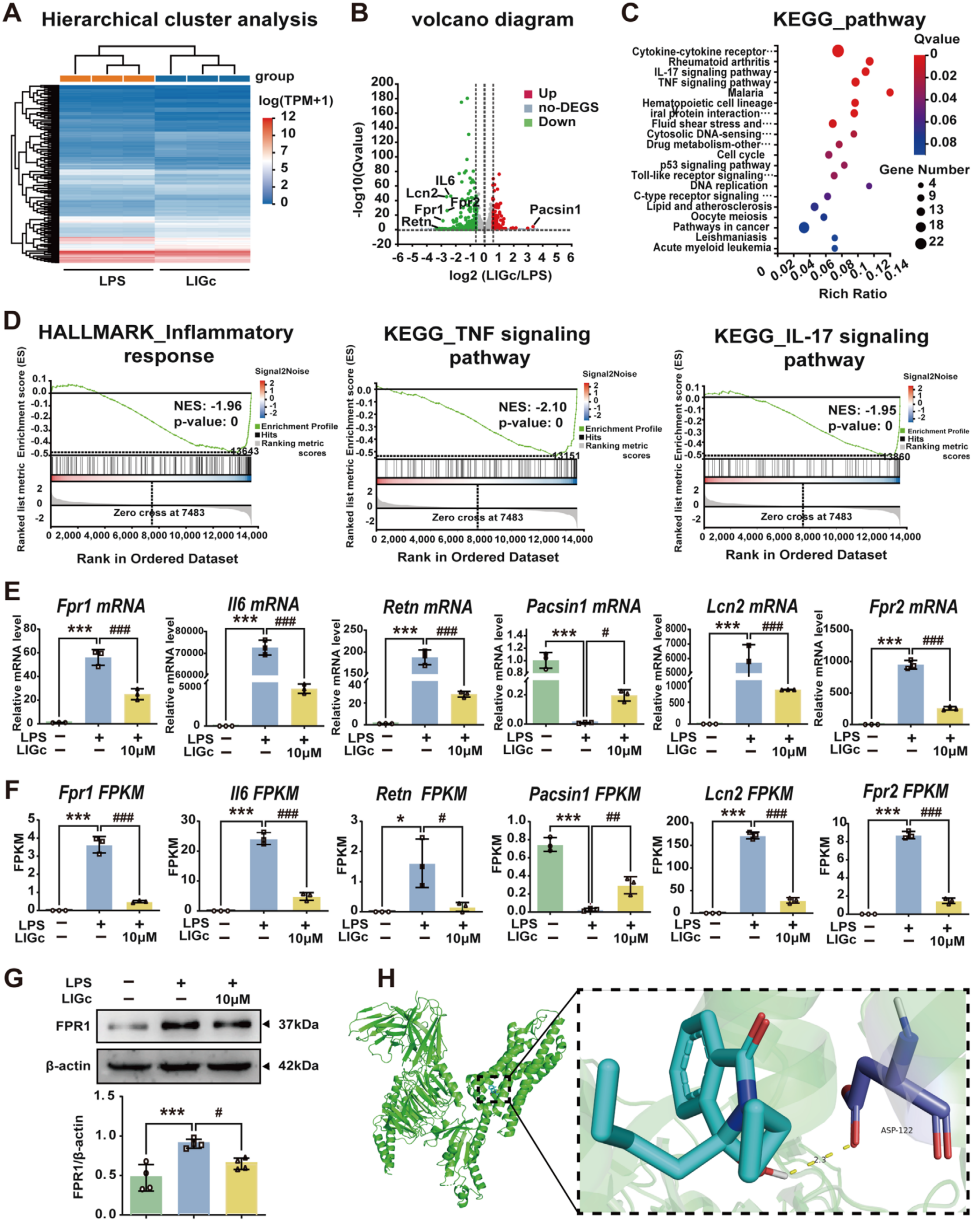
Transcriptomic analysis indicates that the anti‐inflammatory effect of LIGc is associated with the *Fpr1* gene. (A) Hierarchical cluster diagram of RNA‐seq data. (B) Volcano plot illustrating DEGs between the LPS‐ and LIGc‐treated groups. The vertical gray line denotes a fold change of 1.5, and the horizontal gray line indicates an adjusted *p* value of 0.05. (C) Analysis of KEGG signaling pathways enriched with DEGs pre‐ and post‐LIGc treatment. Bubble colors denote adjusted *p* values, with bubble sizes corresponding to the number of enriched genes in each pathway. (D) GSEA assessing enrichment of DEGs in inflammation‐related signaling pathways. (E) Validation of DEG expression via qRT‐PCR from RNA‐seq data. Results are presented as mean ± SD (*n* = 3 independent experiments). Compared to the control group, ***p < 0.001; compared to the LPS group, #*p* < 0.05, ###*p* < 0.001. (F) Statistical analysis of DEG expression from RNA‐seq data. Compared to the control group, *p < 0.05, ***p < 0.001; compared to the LPS group, #*p* < 0.05, ##*p* < 0.01, ###*p* < 0.001. (G) Western blot analysis of FPR1 protein expression in RAW264.7 cells pre‐ and post‐LIGc treatment. Results are expressed as mean ± SD (*n* = 4 independent experiments). Compared to the control group, ***p < 0.001; compared to the LPS group, #*p* < 0.05. (H) Three‐dimensional diagram illustrating molecular docking interactions between LIGc and FPR1.

To further delineate the anti‐inflammatory mechanisms of LIGc, RNA‐seq technology was employed to analyze its impact on the transcriptional profile in LPS‐stimulated RAW264.7 cells. DEGs were identified across the Control, LPS, and LIGc‐treated groups using criteria of fold change (≥ 1.5) and adjusted *p* value (≤ 0.05). Hierarchical clustering heatmaps and volcano plots revealed 4399 DEGs in the Control and LPS groups, with 2209 upregulated and 2190 downregulated genes (Figure S4A,B). In the LIGc‐treated group compared to the LPS group, 342 DEGs were identified, consisting of 161 upregulated and 181 downregulated genes (Figure 5A,B), indicating distinct expression profiles between these groups. To systematically analyze the regulatory effects of LIGc on DEGs, we conducted bio‐functional and signaling pathway enrichment analyses. GO enrichment analysis (Figure S4C) and KEGG pathway analysis (Figure 5C) revealed that these DEGs are intimately associated with immune responses and inflammatory signaling pathways. GSEA method demonstrated that LIGc significantly suppressed multiple inflammation‐related KEGG and HALLMARK pathways, including the IL‐17 signaling pathway, TNF‐α signaling pathway, Toll‐like receptor signaling pathway, IL‐2‐STAT5 signaling pathway, and JAK‐STAT3 signaling pathway (Figure 5D, Figure S3D), thereby closely correlating with its anti‐inflammatory effect. Functional analysis of these DEGs suggests that LIGc may mitigate pro‐inflammatory responses by modulating the transcriptional activity of specific genes related to inflammation. Subsequently, we conducted a screening process and validated the aforementioned genes in RAW264.7 cells using qRT‐PCR. Initially, we selected six DEGs with significant changes before and after LIGc intervention—*Fpr1*, *Il6*, *Retn*, *Pacsin1*, *Lcn2*, and *Fpr2*—for qRT‐PCR verification, as shown in Figure 5E. The results were consistent with the RNA‐seq analysis, as depicted in Figure 5F. Among these DEGs, it has been reported that the upregulation of FPR1 following ischemic stroke is closely associated with secondary neurological deficits and neuroinflammatory responses. FPR1 antagonists have been shown to inhibit peripheral monocyte migration into the brain, thereby ameliorating neuroinflammation post‐ischemic stroke [38]. Furthermore, elevated FPR1 expression in microglia can induce dopaminergic neuron loss via pro‐inflammatory factors release, whereas FPR1 antagonists can reverse these effects [24]. Interestingly, qRT‐PCR and western blot analyses revealed that LIGc significantly suppressed both gene and protein expression of FPR1 in LPS‐induced RAW264.7 cells (Figure 5E,G). Additionally, molecular docking results further suggested that LIGc could form hydrogen bonds with FPR1, exhibiting favorable binding activity with a binding energy of −6.3 kcal/mol (< −5 kcal/mol), as shown in Figure 5H. These findings indicate that LIGc may exert its anti‐inflammatory effects by downregulating the levels of FPR1.

### 3.6 LIGc Inhibits Pro‐Inflammatory Activation of Microglia/Macrophages by Downregulating FPR1 In Vitro

**FIGURE 6:**
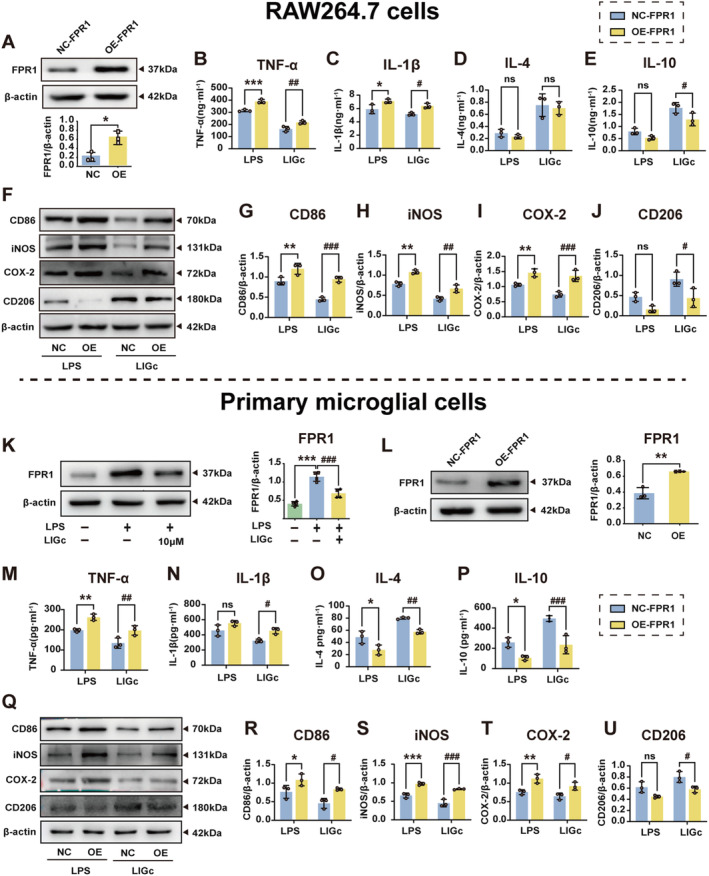
LIGc diminishes the inflammatory response in RAW264.7 cells and primary microglia by downregulating FPR1 expression. (A, L) Western blot analysis was employed to detect FPR1 overexpression in both RAW264.7 cells and primary microglia. Compared to the empty adenovirus (NC‐FPR1) group, *p < 0.05, **p < 0.01. (B–E, M–P) ELISA was utilized to assess the secretion of pro‐inflammatory mediators TNF‐α and IL‐1β, as well as anti‐inflammatory cytokines IL‐4 and IL‐10 in RAW264.7 cells and primary microglia. (F–J, Q–U) Western blot analysis was conducted to evaluate the expression of inflammation‐related proteins in RAW264.7 cells and primary microglia. (K) The expression of FPR1 protein in primary microglia following LIGc treatment was specifically analyzed using western blotting. Results are presented as mean ± SD (*n* ≥ 3 independent experiments). Compared to the LPS‐NC group, *p < 0.05, **p < 0.01, ***p < 0.001; compared to the LIGc‐NC group, #*p* < 0.05, ##*p* < 0.01, ###*p* < 0.001.

Given the regulatory influence of LIGc on FPR1 in RAW264.7 cells, we extended our investigation to primary microglia. As shown in Figure 6K, LPS stimulation significantly elevated FPR1 protein expression in microglia, which was subsequently reversed by LIGc treatment. To ascertain whether LIGc's anti‐inflammatory properties are mediated through FPR1, we employed RAW264.7 cells and primary mouse microglia engineered to overexpress FPR1. The efficiency of ADV4‐*Fpr1* infection was confirmed using western blot analysis 72 h postinfection (Figure 6A,L). Both ELISA and western blot analyses revealed that FPR1 overexpression substantially negated the inhibitory effects of LIGc on LPS‐induced pro‐inflammatory markers in RAW264.7 cells, including TNF‐α (Figure 6B), IL‐1β (Figure 6C), CD86 (Figure 6F,G), iNOS (Figure 6F,H), and COX‐2 (Figure 6F,I). Furthermore, FPR1 overexpression diminished the upregulation of anti‐inflammatory markers induced by LIGc, such as IL‐4 (Figure 6D), IL‐10 (Figure 6E), and CD206 (Figure 6F,J). Comparable results were observed in primary microglia, where FPR1 overexpression reversed the anti‐inflammatory impact of LIGc (Figure 6M–U). These results collectively suggest that LIGc exerts significant anti‐inflammatory effects against LPS‐induced neuroinflammation in vitro by specifically targeting FPR1 expression.

### 3.7 Activation of the FPR1/NLRP3 Signaling Axis Reverses the Anti‐Inflammatory Effect of LIGc In Vitro

**FIGURE 7:**
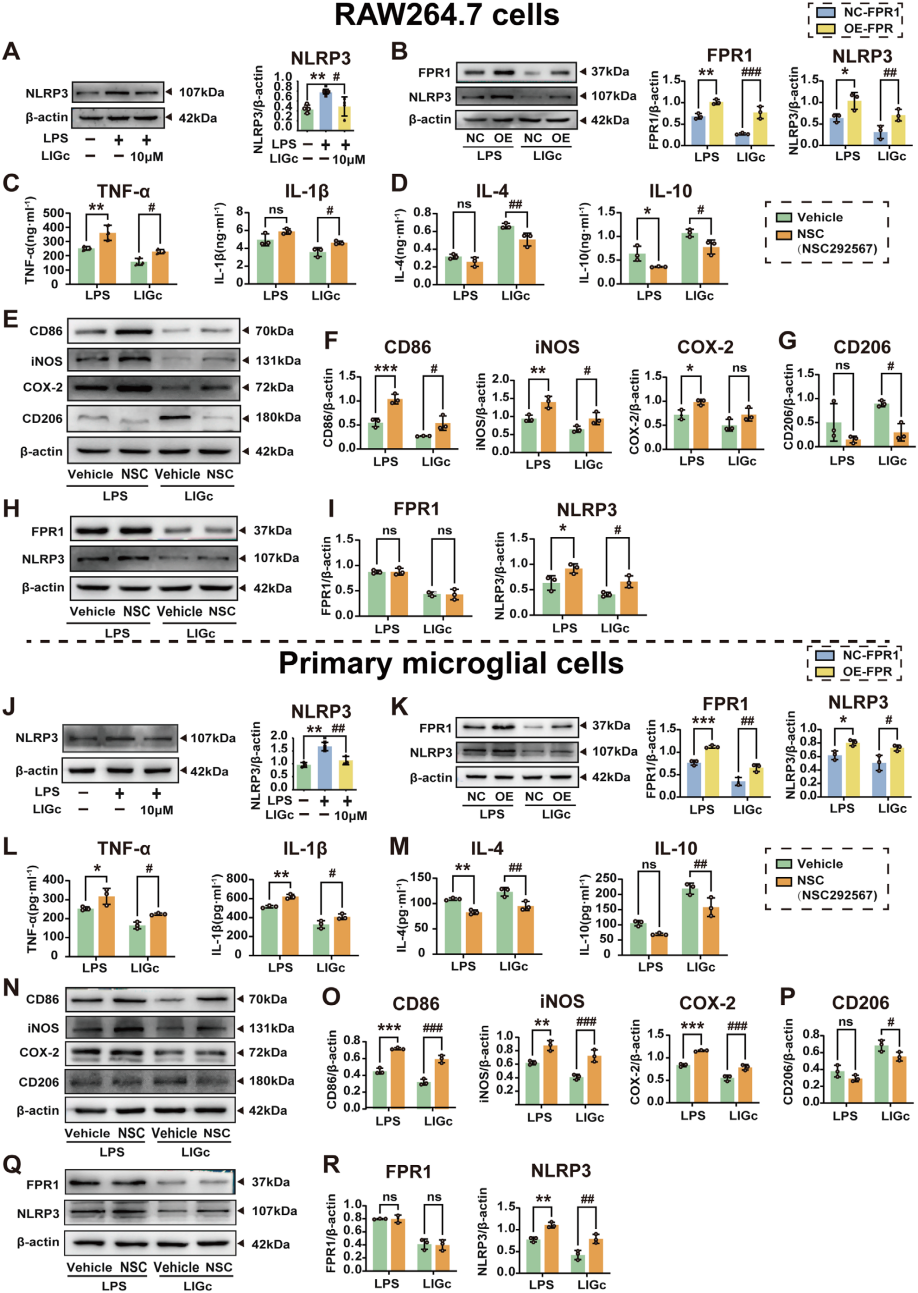
LIGc targets the FPR1/NLRP3 signaling axis to suppress inflammatory responses in both RAW264.7 cells and primary microglia. (A, J) The effect of LIGc intervention on NLRP3 expression was assessed in LPS‐stimulated RAW264.7 cells and primary microglia. Results are presented as mean ± SD (*n* ≥ 3 independent experiments). Compared to the control group, **p < 0.01; compared to the LPS group, #*p* < 0.05, ##*p* < 0.01. (B, K) The impact of FPR1 overexpression on NLRP3 expression in RAW264.7 cells and primary microglia was investigated. (C, D, L, M) ELISA was utilized to quantify the production of pro‐inflammatory mediators TNF‐α and IL‐1β, as well as anti‐inflammatory cytokines IL‐4 and IL‐10 in RAW264.7 cells and primary microglia. (E–G, N–P) Western blot analysis was conducted to assess the expression of inflammation‐related proteins in RAW264.7 cells and primary microglia. (H, I, Q, R) Western blot analysis was performed to examine the expression levels of FPR1 and NLRP3 proteins in RAW264.7 cells and primary microglia. Results are expressed as mean ± SD (*n* = 3 independent experiments). Compared to the LPS‐NC/LPS‐Vehicle group, *p < 0.05, **p < 0.01, ***p < 0.001; Compared to the LIGc‐NC/LIGc‐Vehicle group, #*p* < 0.05, ##*p* < 0.01, ###*p* < 0.001.

Previous studies have demonstrated that activation of FPR1 promotes the expression of microglial inflammatory factors through NLRP3 inflammasome activation, leading to mitochondrial oxidative stress in dopaminergic neurons and subsequent neuronal apoptosis [24]. Conversely, deletion of the *Fpr1* gene impedes NF‐κB nuclear translocation, thereby attenuating the NLRP3 inflammasome and mitogen‐activated protein kinase (MAPK) signaling pathways [25]. To investigate whether LIGc's anti‐inflammatory effects via FPR1 are mediated through the NLRP3 inflammasome, we utilized western blot analysis to examine the involvement of NLRP3 inflammasomes in FPR1‐mediated inflammation and the anti‐inflammatory action of LIGc. Our findings indicate that LIGc treatment downregulates NLRP3 expression in LPS‐induced RAW264.7 cells and primary microglia (Figure 7A,J), while FPR1 overexpression reverses this effect (Figure 7B,K).

Furthermore, to ascertain the pivotal role of the NLRP3 inflammasome in LIGc‐mediated anti‐inflammatory responses, RAW264.7 cells and primary microglia were treated with an NLRP3 agonist (NSC292567), followed by the assessment of changes in pro‐inflammatory and anti‐inflammatory markers. ELISA and western blot analyses revealed that compared to the LPS group, LIGc treatment significantly suppressed pro‐inflammatory responses in RAW264.7 cells, whereas NSC292567 attenuated the LIGc‐induced reduction in TNF‐α (Figure 7C), IL‐1β (Figure 7C), CD86 (Figure 7E,F), iNOS (Figure 7E,F), and COX‐2 (Figure 7E,F) levels, and reversed the increase in IL‐4 (Figure 7D), IL‐10 (Figure 7D), and the anti‐inflammatory protein CD206 (Figure 7E,G) expression. Importantly, NSC292567 did not significantly alter FPR1 expression (Figure 7H,I), suggesting that NLRP3 operates downstream of FPR1 post‐LIGc treatment. These observations were corroborated in primary mouse microglia (Figure 7L–R). Our findings collectively underscore the in vitro anti‐inflammatory capabilities of LIGc, which are achieved by targeting the NLRP3 inflammasome and modulating the FPR1/NLRP3 signaling pathway.

### 3.8 LIGc's Modulation of the FPR1/NLRP3 Pathway in Neuroinflammation and tMCAO Models

**FIGURE 8:**
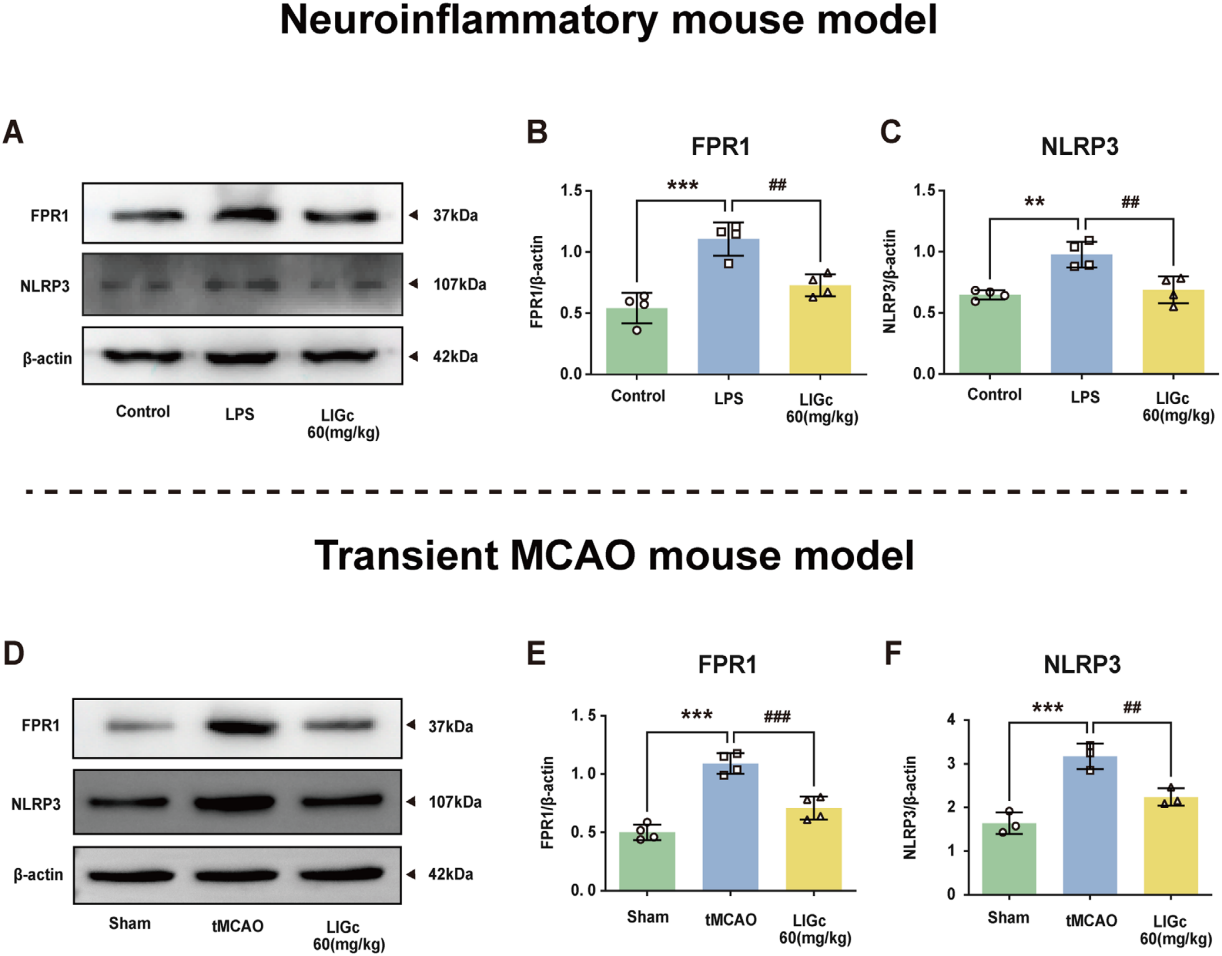
The anti‐inflammatory effect of LIGc in neuroinflammatory and tMCAO mice is potentially mediated through the modulation of the FPR1/NLRP3 signaling pathway. (A–C) Western blot analysis was performed to assess the expression of FPR1 and NLRP3 proteins in the cerebral cortex of neuro‐inflammatory mice. Compared to the Control group, **p < 0.01, ***p < 0.001; compared to the LPS group, ##*p* < 0.01. (D–F) Western blot analysis was conducted to measure the expression levels of FPR1 and NLRP3 proteins in brain tissues of tMCAO mice (3 mice per group; *n* = 4 independent experiments). Results are presented as mean ± SD. Compared to the Sham group, ***p < 0.001; compared to the tMCAO group, ##*p* < 0.01, ###*p* < 0.001.

Finally, we explored the involvement of the FPR1/NLRP3 axis in both LPS‐induced neuroinflammation and ischemic stroke models in mice. Consistent with our in vitro findings, we observed a significant increase in the expression of FPR1 and NLRP3 in the cerebral cortex of LPS‐treated mice, which was effectively reversed by LIGc treatment (Figure 8A–C). Furthermore, in mice subjected to tMCAO, there was notable upregulation of FPR1 and NLRP3 expression in the cerebral cortex, which was attenuated by LIGc administration (Figure 8D,E). These results indicate that LIGc markedly suppresses the inflammatory response in the cerebral cortex of mice with neuroinflammation and ischemic stroke by modulating the FPR1/NLRP3 signaling pathway, thereby ameliorating their neurological deficits and disease progression.

## 4 Discussion

Microglia/macrophage‐mediated inflammatory cascades exacerbate cerebral ischemic injury and are associated with motor and cognitive impairments following stroke [39, 40]. In recent years, strategies aimed at modulating microglia/macrophage activation toward an anti‐inflammatory phenotype to mitigate secondary neurological damage have emerged as a research focus in the treatment of ischemic stroke [14, 41]. Increasing evidence indicates that extracellular administration of certain cytokines and drugs can induce microglia/macrophages to adopt an anti‐inflammatory state [42, 43]. Particularly, the identification of novel drugs targeting the anti‐inflammatory phenotype of microglia/macrophages has provided a foundation for targeted therapeutic approaches in ischemic stroke [44]. LIGc, with a chemical structure similar to that of butylphthalide, is a stable chiral cyclopropiolactam derivative derived from ligustilide [29]. This new monomeric derivative exhibits significantly enhanced oral bioavailability, facilitates penetration of the blood–brain barrier, maintains stable chemical properties, and demonstrates favorable safety characteristics [29]. In our previous studies, LIGc was observed to attenuate LPS‐induced activation of the NF‐κB signaling pathway in BV2 cells, thereby inhibiting the production of pro‐inflammatory cytokines and mitigating the neuroinflammation mediated by BV2 cells [30]. Furthermore, LIGc was found to mitigate the inflammatory response of chondrocytes and reduce chondrocyte apoptosis [45]. These collective findings suggest that LIGc holds promise as a potential treatment for neuroinflammatory conditions such as ischemic stroke.

**FIGURE 9:**
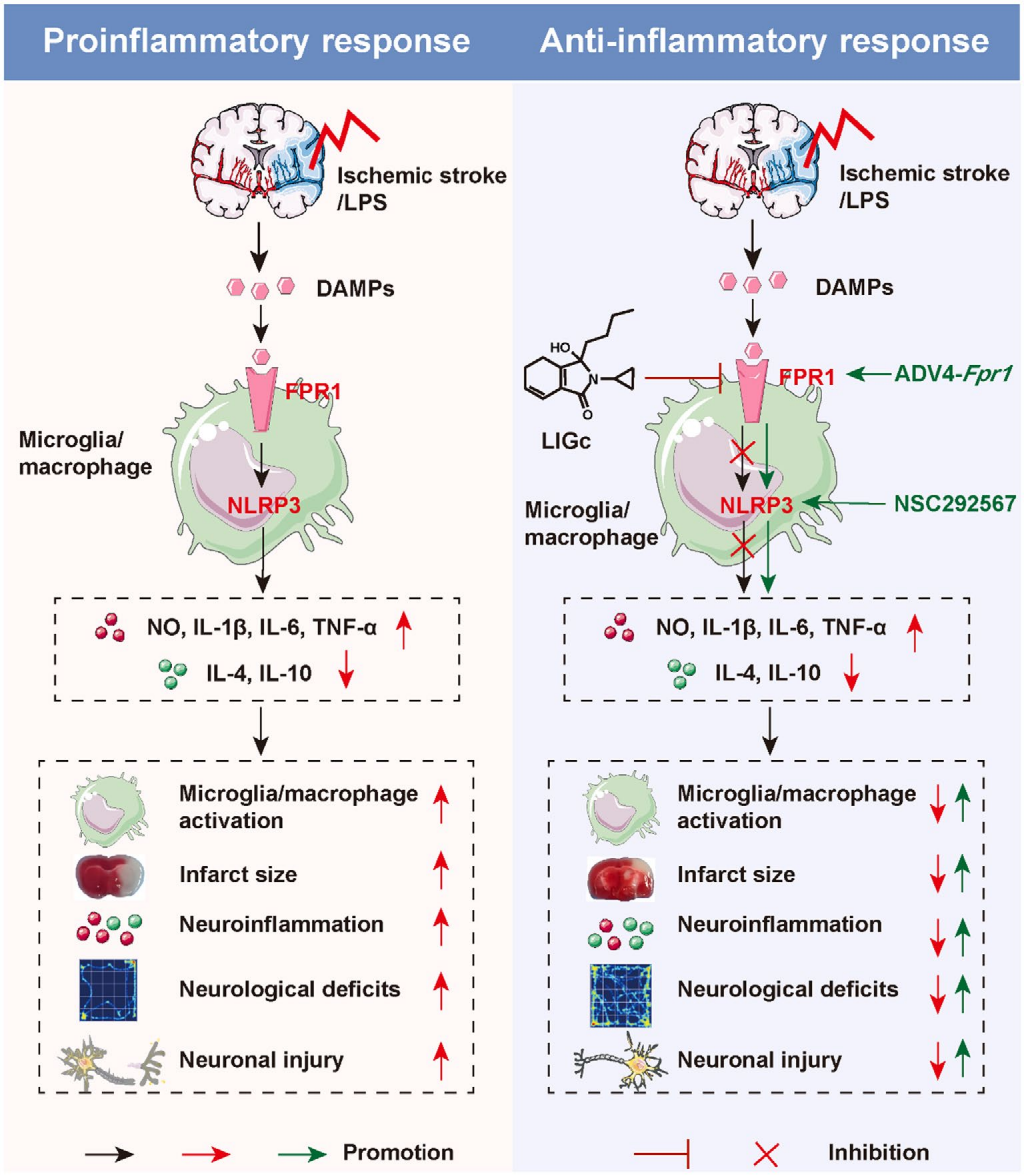
The anti‐inflammatory and neuroprotective mechanisms of the novel drug LIGc in models of neuroinflammation and cerebral ischemic injury involve its ability to downregulate the expression of the FPR1/NLRP3 signaling pathway in microglia/macrophages. This regulation facilitates the transformation of these cells into an anti‐inflammatory phenotype, leading to inhibition of pro‐inflammatory factor release and promotion of anti‐inflammatory factor production.

In this study, we employed the RAW264.7 cell model, primary mouse microglia model, a mouse model of neuroinflammation, and a model of cerebral ischemic injury to investigate the role of LIGc in neuroinflammation following ischemic stroke. Our research has yielded several novel findings: (1) The novel monomer derivative of traditional Chinese medicine, LIGc, exhibits inhibitory effects on the release of pro‐inflammatory factors triggered by LPS in RAW264.7 cells and primary mouse microglia, while concurrently promoting the production of anti‐inflammatory factors. This action facilitates the transition of microglia/macrophages toward an anti‐inflammatory phenotype; (2) In mouse models of neuroinflammation induced by LPS and focal I/R injury, LIGc intervention significantly reduces pro‐inflammatory responses in the brain tissue, resulting in improvements in motor function and neurological deficits; (3) RNA‐seq analysis revealed that *Fpr1* is a potential key target gene for LIGc's anti‐inflammatory effects. Specifically, FPR1 expression was upregulated in LPS‐induced macrophages/primary microglia, LPS‐induced neuroinflammation mouse models, and ischemic/reperfusion mouse brain tissues, which was subsequently suppressed by LIGc intervention; (4) Overexpression of FPR1 or the use of NLRP3 agonists reversed the anti‐inflammatory effects of LIGc in macrophages/primary microglia; and (5) Detailed mechanistic investigations indicate that the aforementioned effects of LIGc are attributed to its ability to downregulate the FPR1/NLRP3 signaling axis, thereby promoting the transition of macrophages/microglia towards an anti‐inflammatory phenotype. The relevant mechanism is schematically depicted in Figure 9.

After ischemic stroke, damage‐related molecular patterns (DAMPs) released by injured neurons activate microglia within the CNS [15]. Concurrently, disruption of the blood–brain barrier facilitates the recruitment and activation of circulating immune cells, such as mononuclear macrophages [46]. Activated microglia/macrophages exhibit functional plasticity in response to various stimuli, demonstrating a dual phenotype that serves dual functions [19, 47]. After ischemic stroke, pro‐inflammatory microglia/macrophages release numerous inflammatory mediators, exacerbating blood–brain barrier damage and augmenting pro‐inflammatory factor release from microglia, while also activating apoptotic molecular mechanisms that induce neuronal cell apoptosis [48]. Conversely, anti‐inflammatory microglia/macrophages secrete cytokines that mitigate the inflammatory milieu and enhance stroke prognosis [49]. Thus, maintaining a balance between these phenotypic transitions is crucial to mitigate the potential harm of sustained, uncontrolled pro‐inflammatory responses [15, 39, 40]. Our research reveals that LIGc significantly inhibits the production of pro‐inflammatory substances, such as NO, TNF‐α, IL‐6, IL‐1β, iNOS, and COX‐2 in LPS‐induced macrophages and primary microglia (Figures 1 and 2). Furthermore, LIGc promotes the production of anti‐inflammatory cytokines IL‐4 and IL‐10 (Figures 1 and 2), indicating its ability to shift microglia/macrophages from a pro‐inflammatory to an anti‐inflammatory phenotype, thereby exerting neuroprotective effects. These findings align with previous studies by He et al. [39] and Yang et al. [47].

Previous studies have demonstrated that intraperitoneal injection of LPS induces neuroinflammation and behavioral dysfunction in mice, likely due to LPS‐mediated blood–brain barrier disruption and subsequent inflammatory response in the brain [39, 50]. In our mouse model of neuroinflammation induced by LPS, we observed motor dysfunction alongside a significant increase in pro‐inflammatory markers in the cerebral cortex. Intervention with LIGc notably ameliorated motor deficits by enhancing the anti‐inflammatory phenotype and suppressing the pro‐inflammatory phenotype (Figure 3), consistent with findings reported by Liao et al. [40]. Additionally, intraperitoneal LPS administration can provoke inflammatory responses in extracerebral organs such as the lungs and liver. The observed behavioral benefits of LIGc in mice may partly stem from its ability to mitigate systemic inflammation. Following ischemic stroke, an imbalance favoring pro‐inflammatory over anti‐inflammatory phenotypes exacerbates infarct expansion and predicts poorer outcomes. Adjusting this balance toward anti‐inflammatory responses holds promise for improving neurological function [40]. Notably, our study demonstrates that LIGc treatment inhibits the pro‐inflammatory phenotype and concurrently promotes the anti‐inflammatory phenotype in the brains of tMCAO mice (Figure 4). Previous research has likewise elucidated analogous findings, where compounds MDL‐811 or Benzoxepane Derivatives ameliorate pathological manifestations in mice by modulating inflammatory phenotype alterations in the brains of neuroinflammatory and tMCAO mice [39, 51]. These findings may serve as a foundation for the potential application of LIGc in the treatment of neuroinflammatory diseases, including ischemic stroke.

To investigate the molecular mechanism by which LIGc influences inflammatory phenotypes, we employed RNA‐seq to systematically analyze the transcriptomic effects of LIGc intervention on LPS‐stimulated RAW264.7 cells. Our results highlight the pivotal role of the pro‐inflammatory response centered around FPR1 in the protective effects mediated by LIGc. Formyl peptide receptors (FPRs) belong to the G protein‐coupled receptors (GPCRs) superfamily [20] and function as pattern recognition receptors that detect pathogen‐associated molecular patterns (PAMPs) [52] or DAMPs [53, 54]. FPR1, the first identified receptor in the FPR family [21], is predominantly expressed in myeloid cells such as macrophages/microglia, dendritic cells, and monocytes [54, 55]. It plays a crucial role in modulating inflammatory responses triggered by microbial infections or tissue damage [22]. Research indicates that FPR1‐mediated microglia/macrophage activation exacerbates the progression of various CNS disorders including epilepsy, cerebral hemorrhage, and multiple sclerosis [56, 57]. In patients with cerebral hemorrhage, microglia near the hematoma express FPR1 and respond to circulating mitochondrial formyl peptides by adopting a pro‐inflammatory phenotype, thereby worsening neurological dysfunction [56]. Additionally, amyloid‐β stimulation of microglial FPR1 leads to activation of the ERK1/2‐MAPK pathway, triggering pro‐inflammatory and oxidative stress responses [58]. Zhou et al. [59] found that silencing FPR1 expression attenuates inflammation, myocardial apoptosis, and ventricular remodeling in rats experiencing myocardial I/R injury, achieved through suppression of the MAPK signaling pathway. Following cerebral ischemia, *Fpr1* ^*−/−*^ mice exhibit reduced infiltration of peripheral monocytes and neutrophils, decreased release of pro‐inflammatory cytokines, and alleviated neuronal injury [38]. In our investigation, we observed that LIGc inhibits the pro‐inflammatory phenotype of microglia/macrophages and promotes an anti‐inflammatory state by downregulating FPR1 expression on microglia/macrophages. Overexpression of FPR1 reversed the anti‐inflammatory effects of LIGc (Figures 5 and 6), aligning with previous findings suggesting that inhibition of pro‐inflammatory mediator release from microglia in *Fpr1* ^*−/−*^ mice presents a potential therapeutic strategy for neuroinflammatory diseases [57].

Furthermore, Wang et al. [24] demonstrated that the FPR1 agonist fMLF stimulates the release of IL‐1β, IL‐6, and TNF‐α by activating NLRP3 inflammasomes in microglia. This inflammatory response can be reversed by administering the FPR1 antagonist HCH6‐1, which exerts neuroprotective effects on dopaminergic neurons. Consistent with this finding, our study revealed that LIGc inhibits the NLRP3 inflammasome level by downregulating FPR1 expression (Figure 7). Notably, overexpression of FPR1 abrogated the inhibitory effect of LIGc on NLRP3 inflammasomes (Figure 7). NLRP3 inflammasomes are pivotal mediators in the inflammatory response, particularly in detecting exogenous pathogens and endogenous cell damage as an essential sensor in the innate immune system [60, 61]. Their critical role in the pathological process of ischemic stroke is well established, and their deficiency or inhibition significantly attenuates ischemic brain injury [62]. Specifically, ischemic brain injury triggers a microglia‐mediated proinflammatory response through the activation of NLRP3 inflammasomes [23, 63]. As a result, the pro‐inflammatory potential of NLRP3 and its involvement in inflammatory diseases render it a prime drug target [64]. In our in vitro studies, we further validated that LIGc exerts anti‐inflammatory effects by inhibiting the expression of NLRP3 inflammasomes in microglia/macrophages, leading to a reduction in the pro‐inflammatory phenotype and an increase in the production of anti‐inflammatory phenotype markers. Notably, NLRP3 agonists were found to abolish these effects (Figures 7 and 8). Interestingly, the administration of NLRP3 agonists did not alter FPR1 expression levels, suggesting that FPR1 acts upstream of NLRP3, and that LIGc's intervention specifically targets the FPR1/NLRP3 signaling pathway to mediate its anti‐inflammatory effects (Figures 7 and 8). This observation aligns with previous research demonstrating that deletion of the *Fpr1* gene significantly downregulates NLRP3 inflammasome signaling and MAPK pathways as well as reduces nuclear translocation of NF‐κB [25], further supporting the critical role of the FPR1/NLRP3 axis in the inflammatory response.

At present, our study faces several limitations. First, our study first confirmed that LIGc can target the FPR1/NLRP3 signaling pathway to modulate the phenotype of microglia/macrophages. This was achieved through in vitro experiments using adenoviral overexpression. Future in vivo experiments will further validate these findings and strengthen our conclusions. Secondly, FPR1/NLRP3 signaling may not represent the sole pathway through which LIGc exerts its anti‐inflammatory effects both in vivo and in vitro. Subsequent studies could leverage RNA‐seq to identify additional targets implicated in LIGc's anti‐inflammatory mechanisms. Finally, this study has primarily examined short‐term effects. Incorporating long‐term follow‐ups will assess the durability of LIGc's benefits and its role in functional recovery, offering a more comprehensive understanding of its therapeutic potential.

## 5 Conclusions

In summary, our study elucidates for the first time that LIGc regulates the inflammatory phenotype of microglia/macrophages by downregulating the FPR1/NLRP3 signaling axis, thus exerting a significant protective effect against cerebral ischemic injury and neuroinflammation. These findings establish a foundation for understanding the molecular mechanisms involved in neuroinflammation during ischemic stroke, identifying therapeutic targets for LIGc, and developing pharmacological interventions to ameliorate neuroinflammatory responses.

## Author Contributions

Juan Gao and Gang Su: conceptualization, methodology, investigation, writing – original draft. Jifei Liu: conceptualization, methodology, formal analysis. Jinyang Song, Wei Chen, and Miao Chai: formal analysis. Xiaodong Xie: investigation. Manxia Wang, Junxi Liu, and Zhenchang Zhang: review and editing; supervision.

## Conflicts of Interest

The authors declare no conflicts of interest.

## Supporting information


Figure S1

## Data Availability

Data that support the findings of this stuy are available upon request.
