# Supplementary material for: A Novel Compound Ligusticum Cycloprolactam Alleviates Neuroinflammation After Ischemic Stroke via the FPR1/NLRP3 Signaling Axis
Source: CNS Neurosci Ther. 2024 Dec 9;30(12):e70158. doi: 10.1111/cns.70158 (PMC11628748; doi:10.1111/cns.70158)
Supplement: Supplementary file 1 — Figure S1 [file CNS-30-e70158-s001.docx]

**Supporting Information**

1. **Supplementary Figure**

**1.1 Supplementary Figure 1**

**
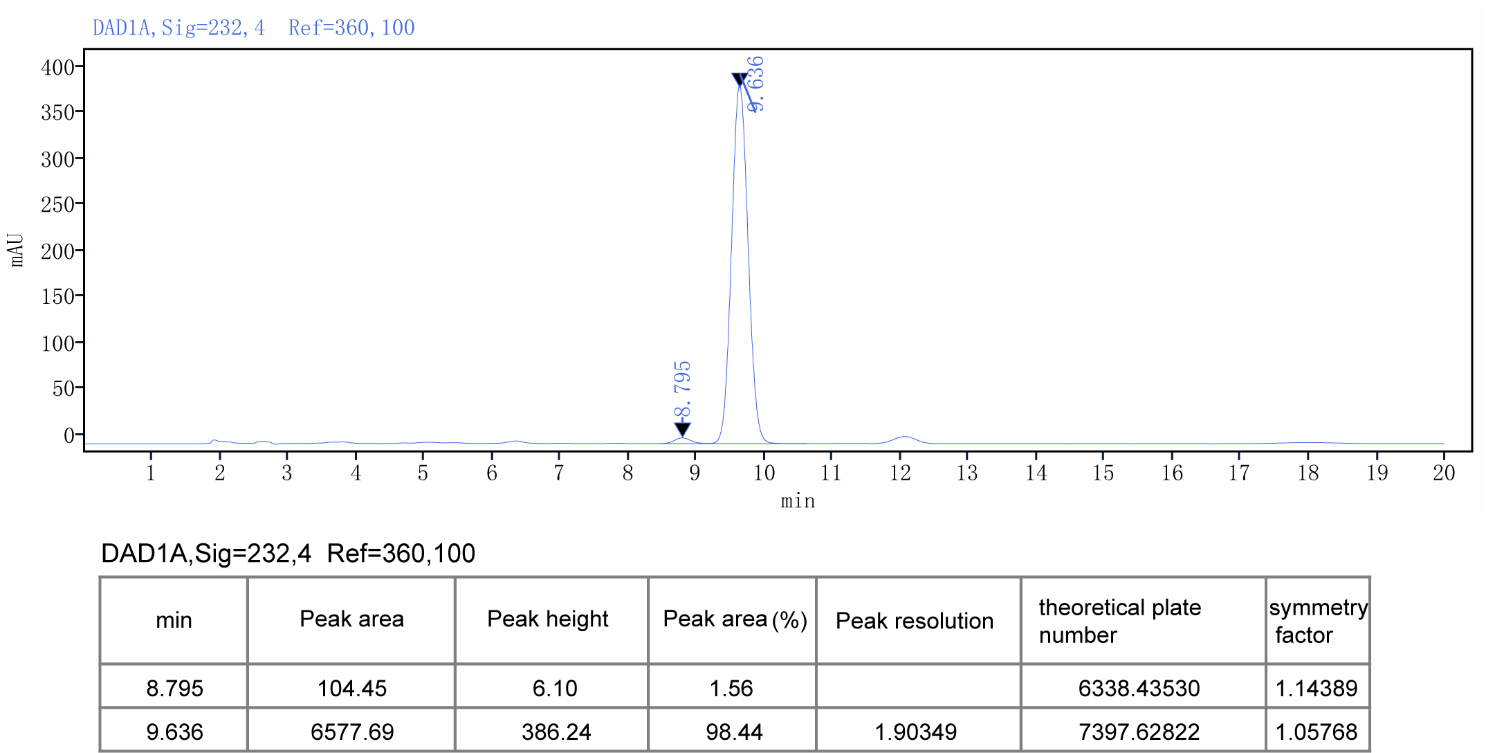
**

**Supplementary Figure 1.** Representative HPLC chromatogram of LIGc.

**Supplementary Figure 2**


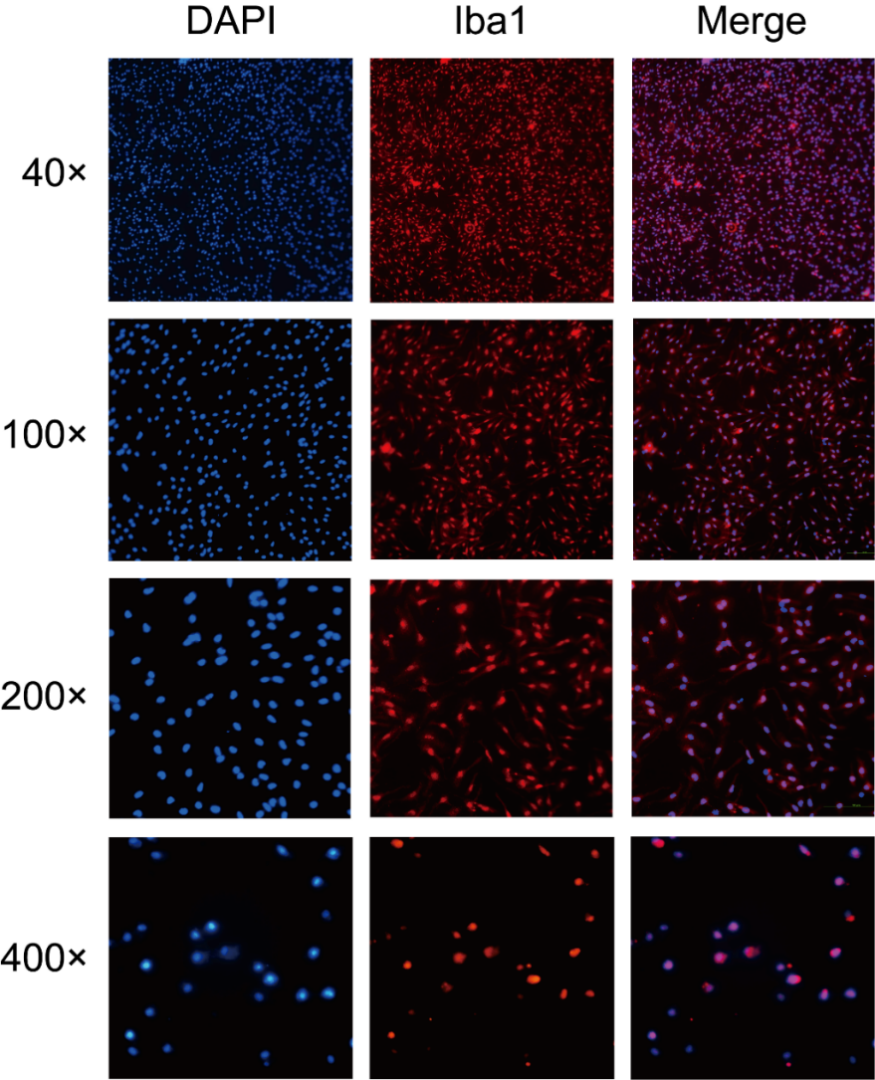


**Supplementary Figure 2.** Immunofluorescent staining of primary mouse microglia.

**1.2 Supplementary Figure 3**


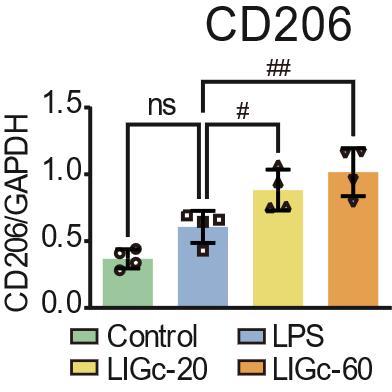


**Supplementary Figure 3.** LIGc ameliorates the neuroinflammatory response in LPS-stimulated mice. The effect of LIGc on the expression of the anti-inflammatory protein CD206 in the brains of mice with neuroinflammation was detected by western blot. Results are presented as mean ± SD (three mice per group; n ≥ 3 independent experiments). Compared to the LPS group, # *p* < 0.05, ## *p* < 0.01.

**1.3 Supplementary Figure 4**


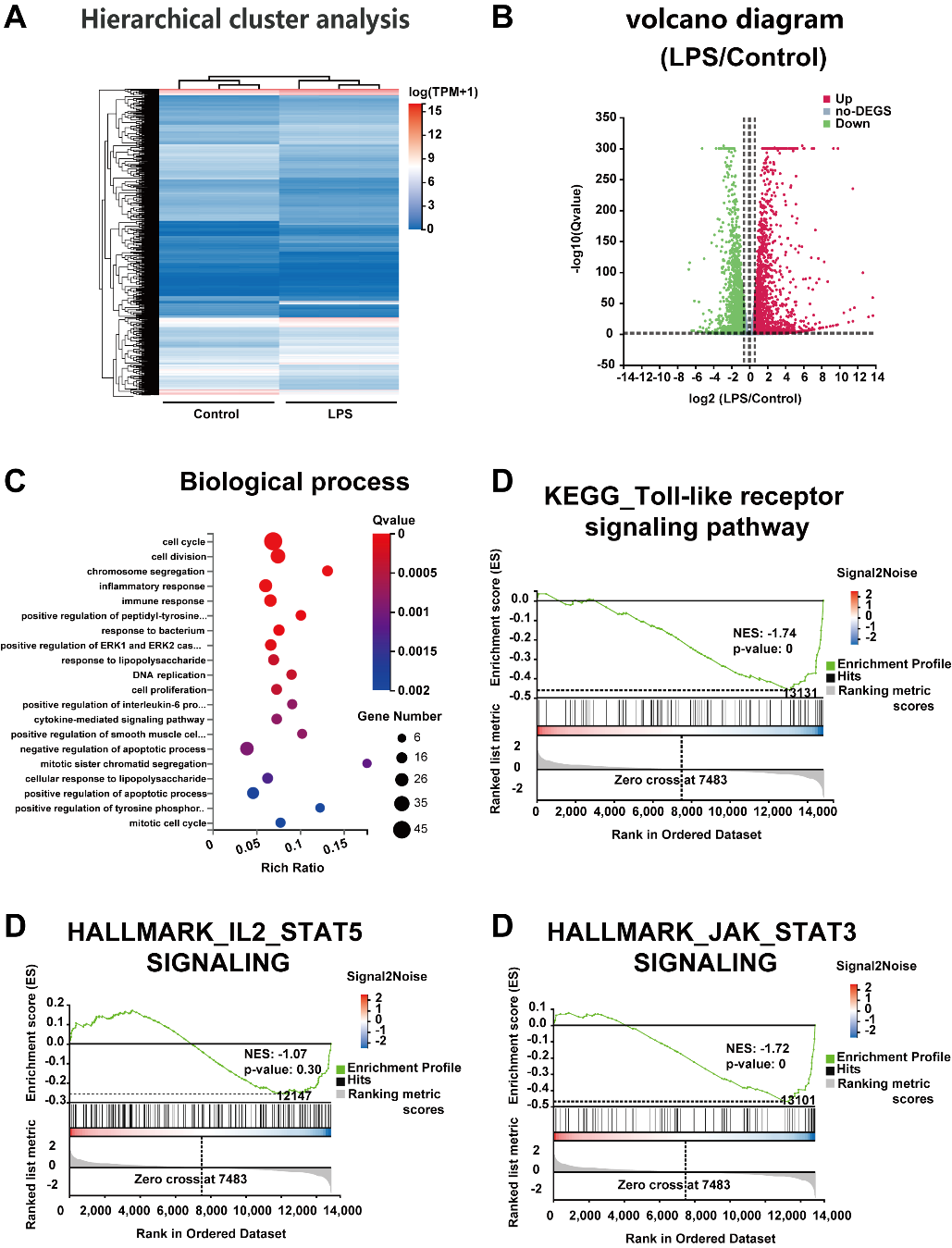


**Supplementary Figure 4.** RNA sequencing results analysis. (A) Hierarchical clustering analysis chart of RNA sequencing. (B) Volcano plot displaying the distribution of differentially expressed genes. The vertical gray lines indicate a fold change of ≥1.5, with red dots representing upregulated genes and green dots representing downregulated genes. (C) GO enrichment analysis of differential genes before and after LIGc treatment. In the chart, bubbles of different colors represent the magnitude of the corrected *p*-values, and the size of the bubbles represents the number of genes enriched in the signaling pathways. (D) GSEA analysis of the enrichment of differential genes in inflammation-related signaling pathways.
